# 淋巴细胞亚群和活化表型在非霍奇金淋巴瘤相关噬血细胞综合征中的表达和诊断价值

**DOI:** 10.3760/cma.j.cn121090-20240409-00129

**Published:** 2024-08

**Authors:** 光丽 尹, 菊娟 王, 甜 田, 丽敏 段, 欣 高, 紫薇 房, 戟 许, 红霞 仇, 磊 范

**Affiliations:** 南京医科大学第一附属医院，江苏省人民医院血液科，南京 210029 Department of Hematology, the First Affiliated Hospital of Nanjing Medical University, Jiangsu Province Hospital, Nanjing 210029, China

**Keywords:** 淋巴瘤，非霍奇金, 噬血细胞综合征, 淋巴细胞, 流式细胞术, 免疫激活, Lymphoma, Non-Hodgkin, Hemophagocytic lymphohistiocytosis, Lymphocyte, Flow cytometry, Immune activation

## Abstract

**目的:**

探讨外周血淋巴细胞和功能活化状态在非霍奇金淋巴瘤相关噬血细胞综合征（NHL-HLH）中的表达和诊断价值。

**方法:**

回顾性分析江苏省人民医院2022年9月至2023年9月收治的30例初诊NHL-HLH患者的临床资料，流式细胞术检测淋巴细胞亚群和功能活化指标。选取同期在本院接受治疗的40例初诊NHL且完善淋巴细胞和功能活化指标的患者作为对照组。比较两组淋巴细胞计数和活化指标的差异。运用受试者工作特征曲线和Logistic回归分析识别NHL-HLH的风险因素。

**结果:**

NHL-HLH组中，T细胞淋巴瘤12例，B细胞淋巴瘤18例。对照组中，T细胞淋巴瘤19例，B细胞淋巴瘤21例。比较两组发现，NHL-HLH组的CD3^+^ T、CD4^+^ T、CD8^+^ T、NK细胞绝对计数低于对照组（*P*值均<0.01）。CD8^+^ T细胞表面CD38和HLA-DR表达水平高于对照组（CD8^+^CD38^+^/CD8^+^ T细胞中位数：57.4％比21.5％，*P*<0.001；CD8^+^HLA-DR^+^/CD8^+^ T细胞中位数49.7％比33.5％，*P*＝0.028）；此外，CD4^+^ T细胞和CD8^+^ T细胞上第二受体信号CD28表达增加（*P*<0.01）。统计分析表明NK细胞绝对计数≤72.0个/µl、CD4^+^CD28^+^/CD4^+^ T细胞>94.2％和CD8^+^CD38^+^/CD8^+^ T细胞>38.4％是预测NHL-HLH发生的危险因素，回归模型的敏感度和特异度分别为86.7％和86.1％，曲线下面积达0.94（*P*<0.001）。

**结论:**

NHL-HLH发生时T淋巴细胞计数减低，而T细胞功能呈活化状态。NK细胞绝对计数≤72.0个/µl、CD4^+^CD28^+^/CD4^+^ T细胞>94.2％和CD8^+^CD38^+^/CD8^+^ T细胞>38.4％是预测NHL-HLH发生的危险因素，可协助临床早期诊疗。

## Abstract (en)

**Objective:**

To determine the expression and diagnostic value of peripheral blood lymphocytes and functional activation status in non-Hodgkin lymphoma with hemophagocytic lymphohistiocytosis（NHL-HLH）.

**Methods:**

We retrospectively analyzed clinical data from 30 newly diagnosed NHL-HLH patients admitted to Jiangsu Province Hospital from September 2022 to September 2023. We assessed peripheral blood lymphocytes and activation status by flow cytometry. Forty newly diagnosed patients with NHL who received treatment at our hospital during the same period and had lymphocyte and functional activation indexes were selected as the control group. The differences in relative and absolute lymphocyte counts and functional activation indexes between the two groups were compared. The optimal cutoff values for continuous variables were calculated from the receiver operating characteristic curve and logistic regression analysis was used to evaluate the risk factors in NHL patients with HLH.

**Results:**

A total of 30 NHL-HLH patients were evaluated, including 12 T-cell lymphoma and 18 B-cell lymphoma patients. Forty individuals were in the control group, which included 19 T-cell lymphoma and 21 B-cell lymphoma patients. The absolute counts of CD3^+^ T, CD4^+^ T, CD8^+^ T, and NK cells, along with the relative count of NK cells, were significantly lower in the HLH group compared with that in the control group（all *P* values<0.01）. The expression of CD38 and HLA-DR on CD8^+^ T-cell activated subgroups was significantly higher in the NHL-HLH group compared with that in the control group（CD8^+^CD38^+^/CD8^+^ T expression median: 57.4％ *vs* 21.5％, *P*<0.001; CD8^+^CD38^+^/CD8^+^ T expression median: 49.7％ *vs* 33.5％, *P*＝0.028, respectively）. In addition, CD28 expression on CD4^+^ and CD8^+^ T cells was significantly higher in NHL-HLH patients（*P*<0.01）. ROC curve and multivariate logistic regression analyses revealed that absolute NK cell count ≤72.0 cells/µl, CD4^+^CD28^+^/CD4^+^ T >94.2％, and CD8^+^CD28^+^/CD8^+^ T >38.4％ were risk factors for predicting the occurrence of NHL-HLH patients. The sensitivity and specificity of the regression model were 86.7％ and 86.1％, respectively, with an area under the curve of 0.94（*P*<0.001）.

**Conclusion:**

In NHL patients with HLH, there was a significant reduction in the absolute number of peripheral blood lymphocyte subpopulations, whereas T-cell function was notably activated. Specifically, absolute counts of NK cells ≤72.0 cells/µl, CD4^+^CD28^+^/CD4^+^ T >94.2％, and CD8^+^CD28^+^/CD8^+^ T >38.4％ were identified as risk factors for predicting the development of NHL-HLH patients. This will assist in early clinical diagnosis and treatment.

噬血细胞性淋巴组织细胞增生症（HLH），也称为噬血细胞综合征（HPS），分为原发性HLH（pHLH）和继发性HLH（sHLH），后者常见因素为感染、自身免疫性疾病和恶性肿瘤等[1]。恶性肿瘤相关HLH多见于B细胞非霍奇金淋巴瘤（NHL）、NK/T细胞NHL，NHL在成人HLH病因中居首位[2]。NHL初次或化疗期间的临床特征与HLH高度重叠，包括发热、血细胞减少、肝脾肿大、铁蛋白升高等[3]。2004年国际组织细胞协会修订的标准（HLH-04）是目前公认的HLH诊断标准，满足八项标准中的五项，则可以诊断HLH。然而，这些实验室指标是基于儿童患者数据得出，缺乏对成人HLH的验证。且需评估NK细胞活性、可溶性CD25浓度、组织噬血现象来确诊是否伴HLH的发生[3]。虽然有研究报道优化HLH炎症（OHI）指数、细胞因子谱升高有助于HLH的预警[4]–[8]。然而，上述检测受耗时、不易获得[9]、易受感染、肿瘤等多种因素影响，且具有未能从HLH发病机制方面阐述病理变化等缺点，因此仍需探索更加优化的HLH检测指标。

HLH的病理机制为各种诱因导致NK细胞和细胞毒性T淋巴细胞（CTL）的过度活化，分泌炎性细胞因子。从病理生理学角度，淋巴细胞亚群及活化状态检测是辅助诊断HLH有价值的工具。前期有文献报道外周血淋巴细胞亚群百分比在HLH中的表达及预后意义[10]–[12]。有文章报道在43例儿童HLH患者中，CD38^+^HLA-DR^+^CD8^+^T细胞指标可作为早期鉴别脓毒症和HLH的最佳标志物[13]。本研究回顾性分析了70例成人NHL患者，采用流式细胞术（FCM）评估淋巴细胞功能亚群及活化状态对非霍奇金淋巴瘤相关噬血细胞综合征（NHL-HLH）的诊断价值，探索其在早期快速辅助诊断NHL-HLH中的应用潜力。

## 病例与方法

1. 病例资料：回顾性分析2022年9月至2023年9月在江苏省人民医院确诊的79例成人HLH患者临床资料，排除非淋巴瘤相关性HLH的32例患者、年龄低于18岁的2例患者以及缺乏完整临床资料的15例患者。最终纳入30例初诊NHL伴HLH作为NHL-HLH组（均满足HLH-04诊断标准；NHL的诊断则基于骨髓穿刺活检、FCM及影像学检查等多种方法综合确定）。另选40例未合并HLH的初诊NHL患者作为对照组。患者均未接受过激素、免疫抑制剂或其他免疫调节剂、生物制剂等治疗。研究经南京医科大学第一附属医院伦理委员会批准（批件号：2019-SR-446）并注册临床试验号（ChiCTR2000032421），已获得查阅患者病历的知情同意。

2. 多参数FCM对淋巴细胞和功能免疫表型分析：取200 µl外周血样本于流式管中，根据试剂说明书用量每管加入相应抗体：① CD45-PerCP-Cy5.5、CD3-FITC、CD4-PE-Cy7、CD8-APC-Cy7、CD16+CD56-PE、CD19-APC；② CD45-PerCP-Cy5.5、CD3-APC-Cy7、CD4-PE-Cy7、CD8-BV421、CD28-PE、HLA-DR-FITC、CD38-APC，涡旋混匀后，室温避光孵育15 min。加入溶血素充分混匀，室温放置10 min，1 500 r/min（离心半径约20 cm），离心5 min后弃上清。加入1 ml磷酸盐缓冲液洗涤，离心后加入500 µl磷酸盐缓冲液上机检测。每管获取不少于50 000个细胞。所用荧光抗体及同型对照均购自美国BD公司。流式细胞仪机型为Beckman Navios（美国Beckman公司），采用Kaluza流式细胞分析软件Version 2.1（美国Beckman公司）分析目标细胞群体的免疫表型特点。门控策略：首先建立FSC/SSC点图，然后在CD45图中选择淋巴细胞，分析CD3阳性T细胞，进一步区分CD4^+^和CD8^+^ T细胞，对CD4^+^ T细胞进行CD28^+^表达分析，对CD8^+^ T细胞进行CD28、HLA-DR、CD38表达的细分分析；同时，在CD19图中选择CD19阳性B细胞；再次分析淋巴细胞门内，进一步区分CD3^−^和CD16^+^CD56^+^的NK细胞。

3. 统计学处理：所有统计分析均使用R 3.6.0版本软件、SPSS 23.0软件进行。计数资料用频率和百分比表示并进行卡方检验，连续变量分布采用Shapiro-Wilk检验进行正态分析，计量资料的两组比较采用Wilcoxon秩和检验。利用受试者工作特征（ROC）曲线计算最佳截断值。使用多因素Logistic回归分析预测NHL-HLH的淋巴细胞功能指标。双侧*P*值<0.05提示差异有统计学意义。

## 结果

1. 基本特征：研究共纳入70例NHL患者，其中NHL-HLH组30例（42.9％），对照组40例（57.1％）。两组患者的临床特征及确诊时的实验室检查结果见表1。两组患者在年龄、性别上差异均无统计学意义（*P*值均>0.05）。

**表1:** 非霍奇金淋巴瘤相关噬血细胞综合征（NHL-HLH）组和对照组患者基本特征

特征	NHL-HLH组（30例）	对照组（40例）	统计量	*P*值
性别（例，男/女）	18/12	23/17	0.044	0.834
年龄［岁，*x*±*s*］	57±17	62±14	2.023	0.139
病理类型			14.480	0.070
T细胞淋巴瘤［例（％）］				
ENKTCL，NT	4（13.3）	8（20.0）		
ANKCL	2（6.7）	0（0）		
PTCL-NOS	2（6.7）	4（10.0）		
AITL	1（3.3）	4（10.0）		
ALCL	0（0）	1（2.5）		
Other T-NHL	3（10.0）	2（5.0）		
B细胞淋巴瘤［例（％）］				
DLBCL	11（36.7）	20（50.0）		
IVLBCL	1（3.3）	1（2.5）		
BCL，NOS	6（20.0）	0（0）		
Ann Arbor分期［例（％）］			16.840	<0.001
Ⅰ～Ⅱ期	0（0）	17（42.5）		
Ⅲ～Ⅳ期	30（100）	23（57.5）		
IG/TCR重排［例（％）］	18（60.0）	13（32.5）	5.254	0.022
复杂核型［例（％）］	13（43.3）	1（2.5）	17.865	<0.001
累及骨髓［例（％）］	27（93.1）	16（40.0）	20.189	<0.001
噬血现象［例（％）］	21（70.0）	4（10.0）	26.880	<0.001
发热≥38.5°C［例（％）］	27（90.0）	6（15.0）	38.698	<0.001
脾肿大［例（％）］	23（76.7）	3（7.5）	38.082	<0.001
肝肿大［例（％）］	5（16.7）	1（2.5）	4.390	0.036
EBV感染［例（％）］	17（56.7）	22（55.0）	0.019	0.890
ANC［×10^9^/L，*M*（范围）］	1.62（0.98～3.93）	3.79（3.12～5.08）	−3.538	<0.001
LYM［×10^9^/L，*M*（范围）］	0.54（0.30～0.86）	1.11（0.96～1.62）	−4.671	<0.001
HGB［g/L，*M*（范围）］	82.3±20.9	111.9±24.3	1.008	<0.001
PLT［×10^9^/L，*M*（范围）］	32（13～69）	232（181～297）	−7.016	<0.001
FIB［g/L，*M*（范围）］	1.48（1.21～2.8）	3.53（2.61～4.71）	−4.848	<0.001
ALT［U/L，*M*（范围）］	30（21～49）	20（12～33）	−2.410	0.016
AST［U/L，*M*（范围）］	73（33～114）	22（17～28）	−4.203	<0.001
LDH［U/L，*M*（范围）］	574（416～1 890）	229（194～325）	−4.482	<0.001
TG［mmol/L，*M*（范围）］	2.2（1.6～2.9）	1.6（1.2～1.9）	−3.278	0.001
ALB［g/L，*M*（范围）］	29.6±6.0	37.4±5.9	0.036	<0.001
IL-6［pg/ml，*M*（范围）］	26.73（9.33～62.67）	11.58（2.76～24.05）	−2.225	0.026
IL-10［pg/ml，*M*（范围）］	151.17（46.74～911.68）	3.13（0.90～10.06）	−5.082	<0.001
IFN-γ［pg/ml，*M*（范围）］	2.35（0.58～7.60）	0.33（0.01～0.62）	−3.846	<0.001
SF［ng/ml，*M*（范围）］	2 899（1 616～5 956）	586（354～825）	−6.967	<0.001

**注** ENKTCL，NT：结外NK/T细胞淋巴瘤，鼻型；ANKCL：侵袭性NK细胞白血病；PTCL-NOS：外周T细胞淋巴瘤-非特指型；AITL：血管免疫母细胞性T细胞淋巴瘤；ALCL：间变性大细胞淋巴瘤；Other T-NHL：其他T细胞非霍奇金淋巴瘤；DLBCL：弥漫大B细胞淋巴瘤；IVLBCL：血管内大B细胞淋巴瘤；BCL，NOS：B细胞淋巴瘤，非特指型；EBV：EB病毒；LYM：淋巴细胞计数；FIB：纤维蛋白原；TG：甘油三酯；ALB：白蛋白；IFN-γ：γ干扰素；SF：铁蛋白

2. NHL-HLH组和对照组患者淋巴细胞计数和活化指标比较：如表2所示，FCM评估初诊NHL-HLH组和对照组患者T、B、NK细胞亚群表达情况，CD3^+^ T、CD4^+^ T、CD8^+^ T细胞相对计数的组间差异均无统计学意义，而NHL-HLH组和对照组B细胞相对计数中位数分别为10.9％和8.0％（*P*＝0.030），NK细胞中位数分别为7.3％和13.3％（*P*＝0.006）。除B细胞绝对计数外，CD3^+^ T、CD4^+^ T、CD8^+^ T、NK细胞绝对计数中位数依次为374个/µl、188个/µl、169个/µl、42个/µl，显著低于对照组的703个/µl、354个/µl、278个/µl、129个/µl（*P*值均<0.01）。

**表2:** 非霍奇金淋巴瘤相关性噬血细胞综合征（NHL-HLH）组和对照组患者外周血淋巴细胞亚群计数比较

细胞亚群	NHL-HLH组（30例）	对照组（40例）	统计量	*P*值
CD3^+^ T细胞比例（%）	72.3（59.8～83.2）	73.1（68.4～81.8）	−0.775	0.438
计数（×10^9^/L）	374.00（192.25～710.00）	735.00（486.50～991.75）	−3.400	0.001
CD4^+^T细胞比例（%）	34.2±12.2	36.5±12.1	0.003	0.437
计数（×10^9^/L）	188.00（100.00～328.25）	376.00（231.25～503.75）	−3.632	<0.001
CD8^+^T细胞比例（%）	32.1±17.3	28.4±10.5	9.010	0.286
计数（×10^9^/L）	169.00（54.25～328.25）	294.00（197.75～459.50）	−2.789	0.005
B细胞比例（%）	10.9（6.7～27.7）	8.0（4.0～11.1）	−2.166	0.030
计数（×10^9^/L）	60.50（25.25～127.50）	78.50（44.00～151.75）	−0.902	0.367
NK细胞比例（%）	7.3（3.2～15.9）	13.3（8.2～21.5）	−2.753	0.006
计数（×10^9^/L）	41.50（11.00～71.93）	129.00（84.50～221.25）	−4.499	<0.001
CD4^+^CD28^+^/CD4^+^T（%）	96.7（95.2～98.2）	90.3（84.6～95.8）	−3.807	<0.001
CD8^+^CD28^+^/CD8^+^T（%）	68.9（46.6～72.3）	49.0（35.3～59.9）	−2.789	0.005
CD8^+^CD38^+^/CD8^+^T（%）	57.4（24.5～76.3）	21.5（11.0～34.5）	−4.136	<0.001
CD8^+^HLADR^+^/CD8^+^T（%）	49.7（26.5～69.6）	33.5（19.2～43.9）	−2.203	0.028

**注** B细胞：CD3^−^CD19^+^；NK细胞：CD3^−^CD16^+^ CD56^+^

NHL-HLH组CD4^+^ T和CD8^+^ T细胞CD28表达比例中位数分别为96.7％和68.9％，均显著高于对照组的90.3％和49.0％（*P*<0.001；*P*＝0.005）。此外，NHL-HLH组中CD8^+^ T细胞CD38和HLA-DR表面激活标志物表达中位数分别为57.4％和49.7％，也显著高于对照组的21.5％和33.5％（*P*<0.001；*P*＝0.028）。

3. 淋巴细胞和活化指标在NHL-HLH辅助诊断中的价值：如表3所示，本研究发现当NK细胞计数≤72个/µl时，诊断NHL-HLH敏感度和特异度均为80.00％，曲线下面积（AUC）为0.82（*P*<0.001）；CD4^+^CD28^+^/CD4^+^ T细胞>94.2％和CD8^+^CD38^+^/CD8^+^ T>38.4％时，对NHL-HLH的敏感度分别为83.33％和70.00％，特异度分别为64.10％和83.78％，AUC为0.77和0.75（*P*值均<0.001）。将淋巴细胞计数和活化指标进行单因素分析（*P*<0.05纳入多因素分析），最终NK细胞计数、CD4^+^CD28^+^/CD4^+^ T细胞和CD8^+^CD38^+^/CD8^+^ T细胞作为分类变量纳入进行分析，结果表明该模型预测NHL-HLH的敏感度为86.7％，特异度为86.1％，AUC为0.94（*P*<0.001）（图1）。

**表3:** 预测非霍奇金淋巴瘤相关噬血细胞综合征变量的截断值和曲线下面积（AUC）

变量	截断值	AUC（95% *CI*）	敏感度（%）	特异度（%）	*P*值
CD3^+^T细胞比例（%）	≤50.5	0.54（0.43～0.67）	20.00	97.44	0.457
CD3^+^CD4^+^ T细胞比例（%）	≤33.2	0.58（0.44～0.71）	60.00	64.10	0.360
CD3^+^CD8^+^ T细胞比例（%）	>36.0	0.53（0.40～0.67）	43.33	83.78	0.283
B细胞比例（%）	>10.3	0.65（0.53～0.76）	53.30	72.50	0.026
NK细胞比例（%）	≤9.0	0.69（0.57～0.80）	66.70	72.50	0.004
CD3^+^T细胞计数（个/µl）	≤282.0	0.74（0.62～0.84）	43.30	97.50	<0.001
CD3^+^CD4^+^ T细胞计数（个/µl）	≤216.0	0.76（0.64～0.85）	66.70	85.00	<0.001
CD3^+^CD8^+^ T细胞计数（个/µl）	≤134.0	0.69（0.57～0.80）	46.70	92.50	0.005
B细胞计数（个/µl）	≤78.0	0.56（0.44～0.68）	70.00	50.00	0.381
NK细胞计数（个/µl）	≤72.0	0.82（0.71～0.90）	80.00	80.00	<0.001
CD4^+^CD28^+^/CD4^+^ T细胞（%）	>94.2	0.77（0.66～0.89）	83.33	64.10	<0.001
CD8^+^CD28^+^/CD8^+^ T细胞（%）	>67.6	0.70（0.57～0.82）	56.67	87.50	0.004
CD8^+^CD38^+^/CD8^+^ T细胞（%）	>38.4	0.75（0.63～0.87）	70.00	83.78	<0.001
CD8^+^HLA-DR^+^/CD8^+^ T细胞（%）	>45.3	0.64（0.51～0.77）	56.67	79.49	0.031

**注** B细胞：CD3^−^CD19^+^；NK细胞：CD3^−^CD16^+^CD56^+^

**图1:**
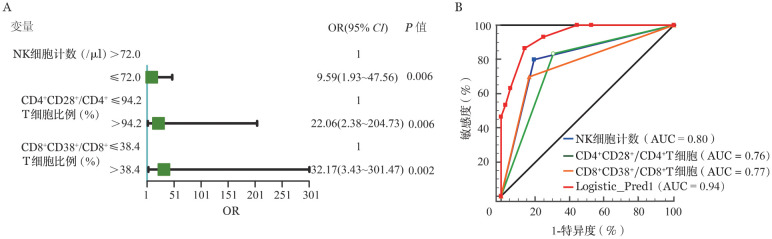
非霍奇金淋巴瘤相关噬血细胞综合征（NHL-HLH）的多因素模型分析 **注** **A** NHL-HLH的多因素Logistic回归分析；**B** Logistic回归模型的受试者工作特征曲线；AUC：曲线下面积

## 讨论

在恶性肿瘤相关性HLH中，淋巴瘤尤其是NHL是最常见的触发因素，通常进展迅速，死亡率高[14]。因此，及时识别出HLH疑似病例并进行快速和早期诊断，对于改善NHL-HLH的预后极为关键。HLH-04标准中的血清可溶性CD25浓度和NK细胞活性检测在常规临床实验室中常常难以执行，等待结果可能会延误HLH的诊疗，增加死亡率[15]。本研究采用多参数FCM进行分析，发现NHL-HLH组NK细胞和T细胞（CD3^+^、CD4^+^、CD8^+^）绝对计数显著降低；此外，CD4^+^CD28^+^/CD4^+^T细胞和CD8^+^CD38^+^/CD8^+^T细胞激活亚群比例升高。基于以上指标多因素联合分析有望早期辅助诊断NHL-HLH（AUC＝0.94）。

在HLH早期阶段，细胞免疫应答在HLH的发病机制中起重要作用，通常能观察到T淋巴细胞数量下降[16]。CD3^+^ T细胞调控免疫应答；CD4^+^ T细胞（辅助性T细胞）在免疫过程中有抗原提呈和促进CD8^+^ T细胞增殖活化作用，为HLH的始动因素；CD8^+^ T细胞包括CTL和抑制性T细胞，其功能不仅包括特异性杀伤靶细胞，还包括分泌抑制因子来削弱或抑制免疫反应，为HLH的关键因素[17]–[18]。与以往许多研究结果一致，我们的研究显示NHL-HLH患者T细胞（CD3^+^、CD4^+^、CD8^+^）绝对计数下降（*P*<0.01）。这表明淋巴细胞亚群的平衡被破坏，T细胞数量的减少可能源于NHL合并HLH时巨噬细胞活化引起的噬血作用、T细胞的衰老和凋亡，或是由于NHL或治疗过程中的感染引起的免疫抑制状态[19]。HLH-04诊断标准增加了对NK细胞活性的评估，即NK细胞活性减少或缺失，该标准尤其针对儿童pHLH的诊断。对于成人HLH患者，Carvelli等[20]研究显示尽管NK细胞表现出活化表型和正常细胞毒性能力，但其数量和产生干扰素-γ（IFN-γ）的能力显著下降。与本研究结果一致，即NHL-HLH患者NK细胞的比例和绝对值均显著降低，有研究在进行HLH动物实验时也发现此类现象[21]。Nichols和Hines[22]认为能够预测HLH发生风险的NK细胞临界值是否存在是一个值得探讨的问题。基于以上，本研究提示NHL患者NK细胞≤72个/µl更易合并HLH，表明NK细胞的水平除了作为HLH的诊断指标，还可以作为NHL伴或不伴HLH的鉴别指标。

除T淋巴细胞数量外，T细胞的功能也是评估疾病严重程度的指标。CD28是T细胞活化过程中必需的协同刺激分子，CD28^+^ T细胞比例反映细胞免疫储备能力，表达下调提示T细胞衰老。CD28^−^ T细胞无法被有效激活、呈“无能”状态。本研究中NHL-HLH患者CD4^+^和CD8^+^ T细胞表面CD28表达水平显著升高（*P*<0.001）。这意味着尽管T细胞总数减少，但仍存在较高比例的T细胞处于激活状态。T细胞活化标志很多，HLA-DR和CD38的表达水平可以反映T细胞的激活状态。多项研究证实HLA-DR和CD38双阳性T细胞比例对疾病进展具有预测价值[23]–[24]。特别是在儿童HLH的研究中，发现CD8^+^ T细胞中CD38/HLA-DR阳性细胞的比例超过7％，对于早期区分HLH和脓毒症具有很高的预测价值，但该研究中并没有涉及到NHL患者[13]。本研究提示NHL-HLH组中CD8^+^T细胞HLA-DR和CD38两个激活标志物表达水平升高，提示异常激活的T细胞在NHL-HLH的发病过程中起到了重要作用。

多项研究表明，尽管没有任何单一的淋巴细胞亚群或表型具备足够的敏感度和特异度来直接用于诊断HLH或淋巴瘤相关HLH[10]–[12]。但综合分析淋巴细胞计数和激活指标可以提供重要信息。本研究中，多因素Logistic回归分析发现基线NK细胞绝对计数、CD4^+^CD28^+^/CD4^+^和CD8^+^CD38^+^/CD8^+^T细胞是NHL-HLH发生的重要预测因素。这些发现提示，NHL合并HLH的发生不仅与T细胞或NK细胞的减少有关，而且与T细胞激活和抑制之间的不平衡有关，需要进一步的研究来深入探究。我们使用多参数FCM对伴或不伴HLH初诊NHL患者进行了淋巴细胞计数及功能活化特征的系统评估。值得注意的是，所有患者在基线检查前未经过任何免疫抑制或其他治疗，以确保结果能准确反映患者淋巴细胞的自然状态。然而，本研究为回顾性分析，虽然已通过年龄和性别匹配的方式尽可能减少其他因素对免疫细胞的影响，但未评估NK细胞活化表型及细胞毒性；由于样本量小，需要未来大样本研究以揭示更多关于NHL-HLH的淋巴细胞免疫特性，从而深入理解淋巴细胞异常如何影响疾病进程。

综上所述，研究结果证实外周血淋巴细胞亚群和功能状态对NHL-HLH有重要的辅助诊断价值。NHL-HLH外周血T和NK细胞绝对计数明显减低，同时T细胞中CD4^+^CD28^+^/CD4^+^ T细胞和CD8^+^CD38^+^/CD8^+^ T细胞激活亚群的比例显著升高。这一发现提示，淋巴细胞计数与活化抗原的表达能够帮助早期识别NHL-HLH，并进行临床干预。
